# Iguratimod: a valuable remedy from the Asia Pacific region for ameliorating autoimmune diseases and protecting bone physiology

**DOI:** 10.1038/s41413-019-0067-6

**Published:** 2019-09-03

**Authors:** Jie Li, Jun Bao, Jian Zeng, Aizhu Yan, Chunqiu Zhao, Qiang Shu

**Affiliations:** 1 Department of Rheumatology, Qilu Hospital of Shandong University, Ji’nan, 250012 Shandong China; 2 The State Key Laboratory of Translational Medicine and Innovative Drug Development, Nanjing, China; 3 Shenzhen Research Institute of Shandong University, Shenzhen, 518057 Guangdong China

**Keywords:** Bone, Multihormonal system disorders

## Abstract

Autoimmune diseases are affected by complex pathophysiology involving several cell types, cytokines, antibodies, and mimicking factors. Different drugs are used to ameliorate these autoimmune reactions, including nonsteroidal anti-inflammatory drugs (NSAIDs), corticosteroids, antiantibodies, and small molecular drugs (DMARDs), and they are clinically in vogue for diseases such as rheumatoid arthritis (RA). Nevertheless, low cost-effectiveness, reduced efficacy, adverse effects, and patient nonresponse are unappealing factors driving the development of new drugs such as iguratimod. Iguratimod is primarily used to ameliorate RA in Japanese and Chinese clinics. However, its efficacy against other autoimmune ailments is also under intense investigation, and the number of investigations is becoming increasingly larger with each passing day. The articular structure comprises synovium, ligaments, and bone. The latter is more complex than the others since it regulates blood cells and autoimmunity in addition to providing skeletal support to the body. Therefore, its protection is also of prime importance in RA and other autoimmune diseases. Herein, we have highlighted the role of iguratimod in autoimmune diseases and bone protection. We suggest that iguratimod’s unique mode of action compared with that of other DMARDs and its good patient response makes it a suitable antirheumatic and bone-protecting drug.

## Introduction

The condition in which an abnormal bodily immune response occurs can be termed an autoimmune disease.^1^ The signs and symptoms range from fever or tiredness to severe inflammation. The National Institute of Health (NIH) has estimated that ~23.5 million Americans are suffering from autoimmune diseases, followed by 22 million from cardiovascular diseases and 9 million from cancer. However, the statistics from the American Autoimmune Related Diseases Association (AARDA) show that ~50 million US citizens are suffering from autoimmune diseases (https://www.aarda.org/). The difference in estimates is because the NIH recognizes only 24 autoimmune diseases based on good epidemiology, whereas the AARDA lists ~80 diseases. However, certain statistics suggest that there are ~100 various diseases caused by autoimmune reactions, with 40 additional ailments having an autoimmune background. Moreover, autoimmune diseases are also listed among the top ten life-threatening maladies for women of all ages around the globe.

Recently, a report demonstrated a 19.1% increase in the rates of autoimmune diseases over the last three decades.^2^ The most common autoimmune diseases include celiac disease, type 1 diabetes, inflammatory bowel disease, multiple sclerosis, psoriasis, systemic lupus erythematosus, and rheumatic diseases, especially rheumatoid arthritis (RA).^3,4^ The latter has been reported with a 7.1% annual increase with a persistently increasing trend. In addition to genetic factors, environmental conditions and demographic locations have been the main factors in the increasing trend of rheumatic diseases.

RA is one of the most important inflammatory diseases that primarily affects the joints of patients. Approximately 1% of the total world population is estimated to be affected by RA, with a higher incidence in women than in men.^5^ Moreover, the lifestyle and genetics of individuals are also risk factors for RA, e.g., smokers are at higher risk than nonsmokers, whereas an individual with *HLA-DR(1, 4,14)* or *QKRAA* genes is also at higher risk.^6^ The etiology of RA, like other autoimmune diseases, is unknown. The patients are maintained on only symptomatic and empirical treatment to reduce their symptoms. These drugs include NSAIDs, e.g., nimesulide and diclofenac, steroids, e.g., prednisolone and dexamethasone, and disease-modifying antirheumatic drugs (DMARDs), e.g., methotrexate (MTX) and anti-IgG antibodies.

Inflammation is well known to be the cellular and vascular response of a body to any tissue insult, and is mostly accompanied by anincreasein temperature at the site, edema, and redness due to extensive cellular activity and enhanced blood flow.^7^ The major cells involved are leukocytes, neutrophils, and antigen-presenting cells (APCs), i.e., macrophages. In the case of RA, the fibroblasts whose primary function is to repair the damaged tissue start working as APCs,^8,9^ leading to further complications of the RA pathophysiology since it results in nonresolving chronic inflammation. RA is accompanied by severe joint pain in the early stages and leads to permanent disability in later stages (~15 years of disease). Small molecular drugs, i.e., DMARDs, including azathioprine, gold, cyclosporine A, MTX, salazosulfapyridine (SASP), and iguratimod (T-614), are more effective than conventional drugs. All of these drugs have clinical importance, and their applications are mostly influenced by physician preferences, patient treatment responses, side effects, and cost-effectiveness.

In this review, we will focus on the latest advances in ameliorating autoimmune diseases and the bone protective effect of iguratimod, the latest DMARD from the Asia Pacific region (i.e., Sino-Japan). Iguratimod has high value as an antirheumatic drug due to its local origin, because most of the drugs developed in the west are trialed and tested on Caucasian individuals and are not equally effective in Asian or African populations.^10^ In Japan, iguratimod has been used in clinical practice since 2012, and its production rights are held by Toyama Chemical Co., whereas its clinical development involves collaborations with Eisai Co. Ltd.^11–14^ Moreover, Jiangsu Simcere Pharmaceutical R&D Co., Ltd. received approval on Aug 25, 2011, for the use of iguratimod in China and they launched “Iremod” as the first commercially available iguratimod preparation on Feb 10, 2012.^15^

### Mechanism of action

**Fig. 1:**
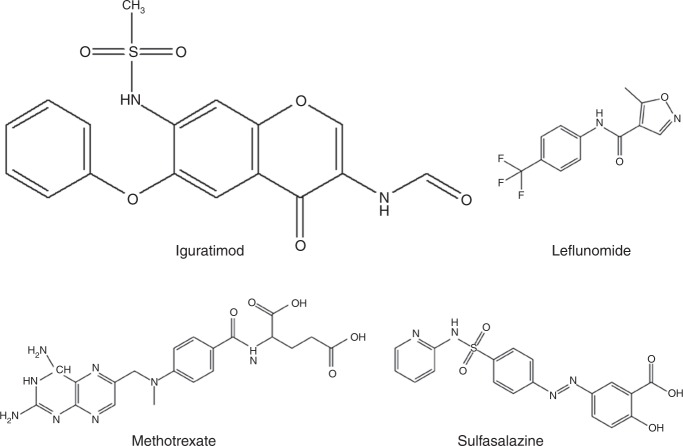
Structural formula of iguratimod and other currently used DMARDs

Iguratimod is a methane sulfonanilide that is chemically composed of (N-[7-[(methanesulfonyle) amino]-4-oxo-6-phenoxy-4H-1-benzopyran-3-yl]-formamide) (Fig. 1). Iguratimod is a small disease-modifying compound that was found to influence several proposed anti-inflammatory and immune-modulatory pathways in experimental models of RA and clinical trials. It has an anabolic effect on the bone metabolism of the infected joint by osteoclastogenesis inhibition and osteoblast differentiation.^16^ Moreover, its downregulatory effect on serum IL-6 levels^17^ provides an edge over conventional NSAIDs, currently in vogue for RA treatment, e.g., nimesulide.^18^ It has also been reported to have lower gastrointestinal ulcerogenic properties, which is the main concern of clinicians regarding NSAIDs that inhibit cyclooxygenase-1 (COX-1) to reduce inflammation. Tanaka et al. demonstrated that iguratimod selectively inhibits COX-2 secretions that lower the levels of prostaglandin E2 (PGE2), a main source of inflammation. Moreover, in two different studies, when cultured fibroblasts were treated with iguratimod, COX-2 mRNA levels^19^ and secretions were reduced, directly affecting the PGE2 inflammatory exudate level and ameliorating inflammation in murine models.^11^

When kaolin-induced murine models were treated with iguratimod, the blood serum profile revealed an elevated level of bradykinin.^13^ Furthermore, due to its small molecular size, iguratimod is orally bioavailable and easily absorbed from the gastrointestinal tract.

Thus far, <30 µg·mL^−1^ of iguratimod has been reported to be experimentally effective in ameliorating inflammation by suppressing various cytokines that are pivotal in initiating inflammatory cascades. Moreover, a clinical dosage of 25 mg twice a day has proven to be efficient and well tolerated, either when administered with food or after fasting, as food does not affect its pharmacokinetics.^20^

There has been strong evidence that iguratimod inhibits nuclear factor-ƙB (NF-ƙB) activity, resulting in reduced antibody production without affecting B-lymphocyte proliferation.^21^

A recent study has suggested that iguratimod suppresses IL-17 in RA synovial fibroblast-like cells, ultimately leading to suppression of inflammation and proinflammatory cytokines regulated via IL-17.^22^ Iguratimod can suppress the mRNA expression of IL-17-related genes and reduce the phosphorylation of mitogen-activated protein kinases (MAPKs). It has been experimentally proven that iguratimod affects Act1, which leads to an Act1-IKKi-TRAF5 gene pathway-induced disturbance of IL-17 in RA fibroblast-like synoviocytes.

### Cytokines

The iguratimod mechanism of action and its efficacy has been widely investigated in various cells involved in inflammatory cascades, especially APCs, i.e., macrophages. Iguratimod has been demonstrated to successfully suppress IL-6, TNF-α, IL-8, IL-1β, and monocyte chemotactic protein 1 (MCP-1) in various inflammatory cell cultures.^23^ The relatively new cytokine IL-17, which is secreted by CD4^+^ T lymphocytes, has been considered equally important to TNF-α, and has been reported as one of the main cytokines in the RA synovial milieu compared with osteoarthritis.^24^ In addition, other T-cell lineages, such as Th1, CD4^+^, and CD25^+^ T-regulatory cells (Treg) and T follicular helper (Tfh) cells, have also been found in the synovium of RA-infected joints.^25^

Iguratimod can significantly decrease the TNF-α concentration.^21,26^ During iguratimod clinical trials, the synovium of RA patients showed decreased production of colony-stimulating factor, IL-6, and IL-8. This suppressive effect has been directly attributed to the downregulation of mRNA expression of these inflammatory cytokines.^14^ However, some studies have also suggested that their comolecules CD106, CD58, and CD54, which are stimulated by INF-ϒ, were also suppressed by iguratimod.^27^

Various studies have reported the anti-inflammatory effect of iguratimod, which significantly suppresses IL-6, IL-8, granulocyte colony-stimulating factor (G-CSF), and granulocyte–macrophage colony-stimulating factor (GM-CSF) in RA synovial fibroblasts;^21^ TNF-α, IL-6, IL-8, MCP-1, and IL-1β from human monocyte THP-1 cells;^26^ and TNF-α from rat alveolar and mouse peritoneal macrophages.^28^

### Antibody production

In certain autoimmune diseases, e.g., RA, a type of Ig called rheumatoid factor (IgG) is increased and used as an important clinical tool for diagnosis and treatment by targeting its activity. Iguratimod has been reported to improve ACR20 response rates for patients with symptomatic clinical RA, and reduce the levels of IgG, IgM, and IgA in patients with RA without affecting the proliferation of B lymphocytes.^29^

Iguratimod has also been reported to induce a reduction in anti-type II collagen antibodies, i.e., IgGa_2_ and IgM, without affecting the IgM concentration in serum.^30^ This antiantibody effect is directly linked to its cartilage and bone destruction prevention effects during the treatment of RA.

### Other DMARDs vs. iguratimod

The other DMARDs—MTX, leflunomide, bucillamine, and sulfasalazine—have been proven to have an excellent anti-inflammatory effect. The European League Against Rheumatism has recommended them as the first line of DMARDs against rheumatic disease, unless contraindicated.^31^ However, prolonged use of MTX and other DMARDs are also coupled with severe adverse effects, such as gastrointestinal reactions and bone marrow suppression.^32,33^ In addition, prolonged use of leflunomide and MTX in autoimmune diseases has been reported to have other side effects and toxicity.^34,35^ Thus far, iguratimod has been reported to have fewer adverse effects, but this finding may be due to its limited clinical approval (only in China and Japan) compared with other DMARDs that are in global use and its inertness in the body.^36^

The efficacy of MTX or other DMARD monotherapy is low in certain RA cases; therefore, its combination therapy with other drugs is favored to ameliorate RA. As a consequence, Ishiguro et al. recently used a combination of iguratimod and MTX for their synergistic effect to ameliorate RA in patients who were nonresponsive to MTX therapy alone during active clinical RA.^37^ These results suggest that 6 mg–8 mg of MTX and 25 mg of iguratimod given on a weekly basis significantly improved the ACR20 by 20% over a 24-week trial. Moreover, other factors, i.e., rheumatic factor, ACR70, ACR50, and disease activity score, were also significantly improved. Due to their different modes of action, a combination of iguratimod and MTX has a stronger synergistic effect.

**Fig. 2:**
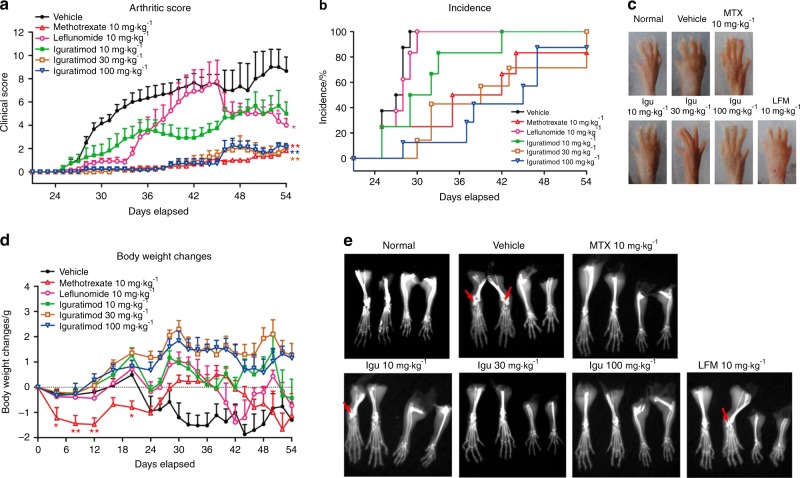
Effects of iguratimod and other DMARDs on collagen-induced arthritis. **a** Arthritis score in different DMARD-treated groups. **b** Arthritis incidence of different treated groups. **c** The extent of paw edema in various groups. **d** The body weight measured in various groups after DMARD treatment. **e** X-ray radiographs of various groups treated with different DMARDs. Arrows indicate the bone erosion area, whereas * and ** indicate significance *P-*values of <0.05 and 0.01 (*P < 0.05, **P < 0.01), respectively. Adapted from Ref. ^21^ copyright © 2013 by The American Association of Immunologists, Inc

Moreover, Luo et al. compared the mode of action of iguratimod with classic RA DMARDs, i.e., MTX and leflunomide, and their study also suggested that iguratimod has a varied mode of action compared with MTX and leflunomide.^22^ Iguratimod directly suppresses mRNA expression of the Act1 gene, leading to disturbances in IL-17 pathways and ultimately reducing inflammation, either directly or by affecting IL-17-associated proinflammatory factors (Fig. 2). In comparison, MTX and leflunomide have been reported to target and affect the TNF-α pathway in inflammatory diseases.^38–40^

In contrast to previous reports, Tokunaga et al. found that sulfasalazine combined with iguratimod and MTX in 16 825 Japanese RA patients revealed no significant difference in monotherapy or combination therapy.^41^ This study included 5 years of clinical data from April 2011 to March 2015. Furthermore, this iguratimod combination therapy in RA patients, who were poor responders to tocilizumab, was found to be quite effective and was recommended as future combination therapy for RA.^42^

### Bone protection

The bones are a key part of the articular anatomy, and are readily affected by autoimmune diseases such as RA. Therefore, the bone structure and its physiological maintenance are also pivotal during RA amelioration. The bone marrow is also reported to be rich in vital hematopoietic stem cells, bone marrow stem cells, adipocytes, and osteocytes. During autoimmune diseases, such as RA, an altered functionality of autoimmune cells (B lymphocytes) may also influence the adjacent cells.^43^ Hence, the bone marrow microenvironment is affected, leading to altered physiology and functionality of bone that ultimately leads to bone erosion and associated conditions.

Similarly, bone marrow mesenchymal stem cells (BM-MSCs) have been reported to directly influence the RA synovium and cells.^44^ Generally, bone loss occurs in three forms, i.e., specific bone loss, erosion, and systemic osteoporosis, and all of them share the same mechanism. Clinical studies of RA patients treated with iguratimod for less than 1 year indicate that most of the focus is on edema, articular structure, and associated pain amelioration.^45^ Therefore, the available bone morphology-associated data are mostly from the bench, and there is a need for full clinical investigations.

The major bone homeostasis and remodeling factors include osteoprotegerin (TNFRSF11B), Dickkopf-1 (DKK-1), receptor activator of nuclear factor–kB (RANK) and RANK ligand (RANKL).^46,47^ In RA, bone erosion is a major associated condition that develops very rapidly. RANKL and macrophage colony-stimulating factor are essential for mimicking osteoclast differentiation. The RANKL has a strong affinity for RANK (i.e., highly expressed on the surface of osteoclast and preosteoclast precursors), and they initiate preosteoclast recruitment and osteoclast activation.

Furthermore, osteoprotegerin is a RANKL decoy receptor that can act as a protective factor used to maintain bone physiology by interacting with RANKL and hampering the RANKL–RANK interaction.^47,48^ The imbalance between the RANKL and osteoprotegerin system results in bone loss and erosion. In addition, the RANKL/osteoprotegerin (OPG) ratio is considered a serum biomarker for clinical investigation of bone loss in RA.^49^ Moreover, the suppression or deletion of RANKL or RANK or enhanced expression of osteoprotegerin has been reported to be associated with severe osteoporotic development, which shows the vital role of these factors in bone physiology and maintenance.^50^

**Fig. 3:**
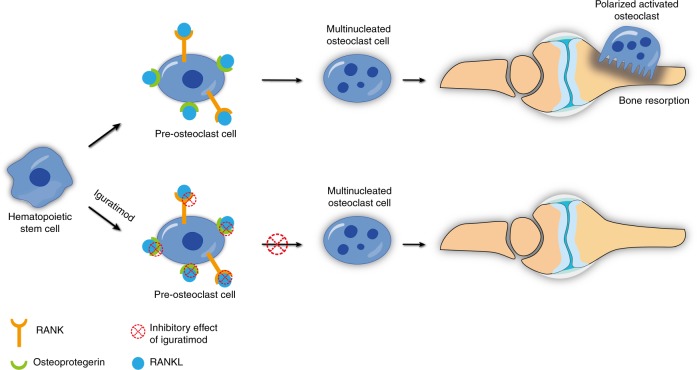
Bone-protecting effect of iguratimod via inhibition of RANKL and osteoprotegerin expression within the bone milieu

Iguratimod has been reported to decrease osteoprotegerin and RANKL, whereas it did not affect DKK-1 after 6 and 12 months in a clinical trial.^51^ Moreover, the effect of iguratimod was not inferior to MTX. In another study, the inhibitory effects of iguratimod alone or in combination with MTX on osteoprotegerin and RANKL were evaluated in RA patients for 12 and 24 weeks after treatment, clinically and in fibroblast-like synoviocyte cultures in vitro.^52^ All investigations revealed that iguratimod suppressed RANKL and osteoprotegerin expression, resulting in a reduced RANKL/OPG ratio. They also showed that the combination of iguratimod and MTX had a synergistic lowering effect on OPG and RANKL that was relatively higher after their individual use (Fig. 3).

During RA, disruption of the synovium results in enhanced cellular activity of B and T lymphocytes, fibroblasts, and proinflammatory cytokines, resulting in a tumor-like structure formation called a pannus.^53^ The pannus invades bone tissue and starts degrading cartilage. Meanwhile, the metalloproteinase-, synovial fibroblast-, and osteoclast inflammation-mediated resistance activity leads to bone and cartilage erosion.^6,54^ It has been reported that 80% of bone erosion occurs within 1 year of RA diagnosis and leads to structural and functional deformity.^55^

Kuriyama et al. have also evaluated the effect of iguratimod on bone structure^56^ and found that in preosteoplastic (MC3T3-E1) and stromal cell lines, osteoblast differentiation to bone formation was stimulated by iguratimod in the presence of bone morphogenetic protein 1 (BMP-1). In addition, the calcium content of mineralized nodules was elevated fourfold after iguratimod treatment. Similarly, osteoclast differentiation was inhibited after iguratimod treatment of the RAW264.7 cell line.^57^ Most recently, Gan et al. reported that iguratimod can significantly inhibit osteoclast differentiation, migration, and bone resorption after activation with RANKL in SRAW264.7 cells.^58^ Iguratimod significantly downregulated osteoclastic gene mRNA, i.e., tartrate-resistant acid phosphatase (TRAP), cathepsin K, and calcitonin receptor, and suppressed the expression of chemokines, such as chemokine C–C-motif ligand-4 (CCL4), CCL7, CCL12, C-Jun, C-Fos, and nuclear factor of activated T-cell cytoplasmic 1 (NFATC1). Moreover, the MAPK and NF-ƙB pathways were also suppressed in the RAW264.7 cell line. These findings strongly indicate that iguratimod not only suppresses inflammatory cytokines in the RA synovial milieu but also assists in bone structure restoration and prevention of resorption.

Moreover, iguratimod suppresses the production of fibroblast-originated collagen formation leading to ossification in RA and protects the bone by activating osteoclasts. Its promotion of osteoblast differentiation can induce osteoblast-specific transcription factors, i.e., osterix, and facilitate bone morphogenetic protein-2 (BMP-2)-mediated bone tissue protection.^29,45^

The cytokines and their mediators present in the bone milieu are the main initiators of a “vicious cycle” that allows tumor cells to interact with osteoblasts, osteoclasts, and bone matrix. Cancer cells that arrive in the bone marrow start secreting cytokines and mediators to make osteoblasts amenable to secreting pro-osteolytic factors that initiate a cascade of events to activate osteoclasts and bone resorption. These activated cells release growth factors that are utilized by cancer cells as growth promoters.^59,60^ Interestingly, the reported cytokine-suppressing effect (e.g., IL-6, NF-ƙB, and IL-17) of iguratimod is beneficial in breaking this vicious cycle in the bone marrow.^61^

**Fig. 4:**
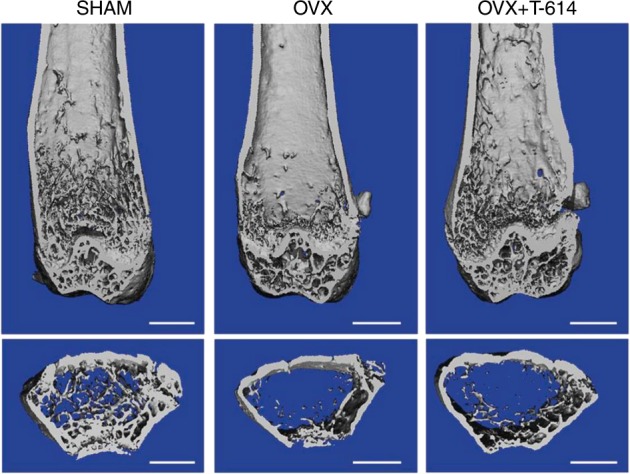
Iguratimod effect on ovariectomized osteoporosis mouse models. The distal femur u-CT images from the Sham-operated group (SHAM), bilateral ovariectomized group (OVX), and OVX-iguratimod-treated groups (OVX + T-164). The scale bar is 1 mm. Adapted from Ref., ^62^ copyright© Wu et al.

In addition to the protective effect of iguratimod on bone resorption during RA, its role in osteoporosis has also been investigated in postmenopausal patients by Wu et al.^62^ They created an osteoporosis model in ovariectomized mice and isolated bone marrow mononuclear cells that were induced to differentiate into osteoclasts with RANKL. After iguratimod treatment, the PPAR-y/c-Fos pathways, which are essential in RANKL-induced osteoclast differentiation pathways, were suppressed, and as a result, trabecular bone loss was significantly attenuated (Fig. 4). These results demonstrate the role of iguratimod in the prevention of osteoclast differentiation, which is of value in clinical applications in postmenopausal osteoporosis patients.

**Fig. 5:**
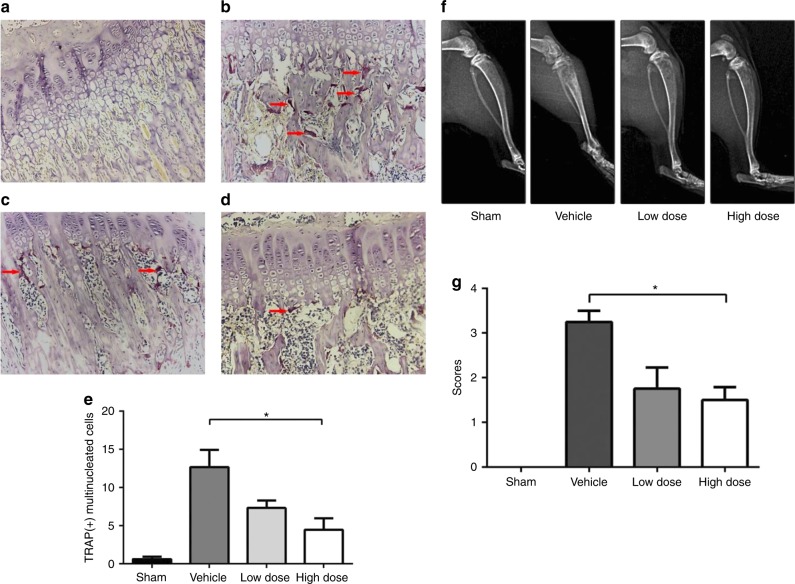
Effect of iguratimod on osteoblast differentiation and bone resorption. **a**–**e** Bone destruction detected by histopathology. Osteoclast in **a** sham (control) group; **b** vehicle group (number of tartrate-resistant acid phosphatase (TRAP) ^+^ cells increased); **c** low --dose iguratimod group (lesser number of TRAP^+^ cells); **d** high-dose iguratimod group (decreased number of TRAP^+^ cells); **e** the number of TRAP^+^ cells in five randomly selected fields at ×400 magnification in each group. **f** X-ray radiographs of rat tibiae in each group. **g** The X-ray score in each group. * indicates a significance *P-*value of less than 0.05. Adapted from Ref., ^63^ copyright© Sun et al.

Iguratimod is an effective bone erosion suppressant not only in RA and other autoimmune diseases but also in cancer-induced bone pain and destruction. Recently, Sun et al. reported the efficacy of iguratimod in a rat model of cancer-induced bone pain as analyzed by mechanical paw withdrawal and pERK and c-Fos measurements in the spinal cord.^63^ In addition, bone destruction was detected by histopathology and X-ray (Fig. 5). They found that pain parameters and bone resorption were improved in the iguratimod-treated group in a dose-dependent manner.

Collectively, the current reports regarding the role of iguratimod in bone erosion suppression, pain reduction, suppression of osteoclast differentiation, and inhibition of the vicious cycle are encouraging, and define its role effectively in bone protection. Moreover, the bone is a key part of the articular anatomy, and has a regulatory role in RA pathophysiology; therefore, iguratimod’s role in RA amelioration is directly proportional to bone protection. Hence, we further discuss the RA ameliorating effect of iguratimod below.

### Rheumatoid arthritis

RA is the primary disease treated with DMARDs. Among the various DMARDs, iguratimod is relatively new and is currently approved for clinical practice only in China and Japan. RA is a chronic systemic inflammatory autoimmune disease that primarily affects the articular joints of patients. The synovial milieu is characterized by an elevated level of T lymphocytes, TNF-α, and other inflammatory factors that result in bone, cartilage, and tendon destruction.^44,64^ The major problem with RA is its chronic nature, which over time (~15 years) leads to severe disability (1.7 million patients in higher income countries and 3.7 million patients in lower income countries) in the population >60 years old.^65^ Moreover, 68% of patients suffer from severe pain, especially in the morning, due to joint stiffness.^66^ Currently, various DMARDs, NSAIDs, TNF-α blockers, interleukins, and CD20 monoclonal antibodies are used to ameliorate RA. In the last decade, a new immunomodulatory small-molecule drug, i.e., iguratimod, has become popular for RA treatment and is more efficient against TNF-α, IL-4, IL-6, NF-ƙB, and IL-17, which are key players in the inflammatory cascade.^30^ Iguratimod can also directly suppress immunoglobulin production in human B lymphocytes without proliferation suppression.^67^ The recent findings of Xu et al. in RA patients in clinical trials have demonstrated that iguratimod can reduce Th17, Th1, and Tfh cells associated with inflammatory cytokines and their transcription factors, while it does not affect Tregs.^25^ Iguratimod has also been reported to successfully improve the disease activity score for C-reactive proteins (CRP) and the erythrocyte sedimentation rate (ESR) in RA patients.^45,68–70^

A recent study by Xu et al. used a combination of iguratimod (25 mg bid) with NSAIDs, i.e., celecoxib 400 mg, to treat RA patients.^36^ In this 12-week trial, the effect was evaluated by the vital parameters of RA, such as inflammatory markers, CRP, anti-citrullinated peptide antibodies, rheumatoid factor, and ESR. This combination of celecoxib and iguratimod significantly suppressed the inflammatory markers in the treated group with subtle to no adverse effects, suggesting that this synergy strongly ameliorated RA during a clinical trial.

Mimori et al. recently determined the real-world safety and efficacy of iguratimod in Japan by following up with 2679 RA patients.^71^ They found that 3.21% of patients had serious adverse effects, 38.41% had adverse effects, and 31.65% had drug-related adverse effects. The authors pronounced iguratimod to be equally safe and clinically efficient as other DMARDs. In another study, Okamura et al. adopted a similar approach ina 52-week follow-up of 41 RA patients using iguratimod to evaluate its efficacy and safety.^72^ The disease activity score, CRP, disease activity indices, and matrix metalloproteinase-3 (MMP-3) were all significantly decreased, and only one case of *Pneumocystis jiroveci* pneumonia was recorded as an adverse effect. Arita et al. also reported that iguratimod given as a 14-week continuous therapy can increase the risk of *P. jiroveci*-associated pneumonia in RA patients.^73^ In addition to biochemical and genetic parameters, 52-week ultrasonography of the joints of RA patients has also been reported, and the efficacy of iguratimod is significant.^74^

The combination therapy of iguratimod with other DMARDs, i.e., MTX, has also been clinically investigated, and was found to have a higher synergistic amelioration effect on RA. Recently, Xia et al. studied 131 Chinese patients with active RA who had previous exposure to other DMARDs.^75^ The authors found that iguratimod combined with MTX had a significant synergistic effect on RA amelioration compared with MTX alone, whereas iguratimod alone was also efficient. The dose used was 25 mg twice a day orally for iguratimod and 10 mg weekly for MTX. In another clinical trial by Duan et al., the same synergistic effect of iguratimod with MTX was reported.^76^ Similarly, Japanese RA patient studies have reported a higher efficacy of iguratimod in combination with MTX.^37,77–79^ After a clinical trial with 123 RA patients, Yoshioka et al. suggested that iguratimod combination or monotherapy were equally efficient and concluded that 12 weeks are long enough period to judge the midterm efficacy of iguratimod.^80^

A recent study from a group of rheumatologists in China has identified the key genes in RA patients responding to iguratimod treatment. They found that patients carrying the *ABCG2 rs2231142* allele were highly responsive to iguratimod, whereas those carrying *NAT2 rs1495742 G* had the lowest response. Furthermore, the CYP2C19*2 rs4244285 A carrier patients had a higher risk of iguratimod toxicity.^81^ This finding not only may help predict the individual’s response to iguratimod but also may be useful in predicting its potential toxicity in RA patients.

Likewise, MMP-1 and MMP-3 levels in RA patients and synovial fibroblast-like cells in vitro have suggested that iguratimod can significantly lower their concentration with clinically and experimentally established doses.^82^ The MMPs actively destroy the cartilage during RA, and their attenuation leads to bone protection.

### Various diseases

#### Inflammatory diseases

Iguratimod has been reported to alleviate autoimmune diseases associated with inflammation. Recently, its applications were further extended to other inflammatory diseases, such as axial spondyloarthritis (axSpA), which is a chronic inflammatory disease primarily affecting the spine and sacroiliac joints.^83^ Luo et al. reported that patients who failed to respond to common NSAIDs were given a 12-week trial of iguratimod with a dosage of 25 mg twice a day.^84^ Although ~50% of patients dropped out due to gastrointestinal adverse effects, those who completed the trial effectively ameliorated their axSpA.

Pulmonary fibrosis is another clinical condition resulting from persistent chronic inflammation leading to alveolitis and pulmonary fibrosis.^85^ Zhao et al. used iguratimod to treat bleomycin-induced pulmonary fibrosis murine models. The histopathological lesions of reduced collagen deposition and alveolar inflammation were observed from day 7 until day 28, and the cytokine levels of IL-1, IL-6, TNF-α, and MMP-9 were significantly lower in the iguratimod group than in the control group.^69^

Various in vivo animal studies have reported that iguratimod can significantly lower the levels of serum monocyte chemotactic protein-1 stimulated by TNF-α injection.^28^ In addition, concanavalin-A-induced hepatitis can be successfully cured by iguratimod injections, which suppress the elevated levels of TNF-α, IFN-_ϒ_, and serum transaminases.^28^

#### Other autoimmune diseases

In addition to the primary use and extensive investigation of iguratimod in RA and associated conditions, other autoimmune disease applications are also encouraging. For instance, inflammatory bowel disease (IBD) is considered an autoimmune inflammatory disease of the gastrointestinal tract characterized by chronic inflammation due to a genetic predisposition, intestinal microflora, and environmental factors.^86,87^ Recently, iguratimod’s role in ameliorating IBD was evaluated in a dextran sulfate sodium-induced murine colitis model by Jiang et al.^88^ They found that 30 mg·kg^−1^ iguratimod given by oral gavage significantly lowered IL-6, TNF-α, and IL-17 levels, whereas it increased IL-10 and transforming growth factor-β (TGF-β) levels. It also downregulated Th17 cells and ROR_ϒ_t and signal transducer and activator of transcription 3 (STAT3) levels, whereas it upregulated Treg cells and STAT5 and FoxP3 transcription factor levels in the intestinal tissue.

**Fig. 6:**
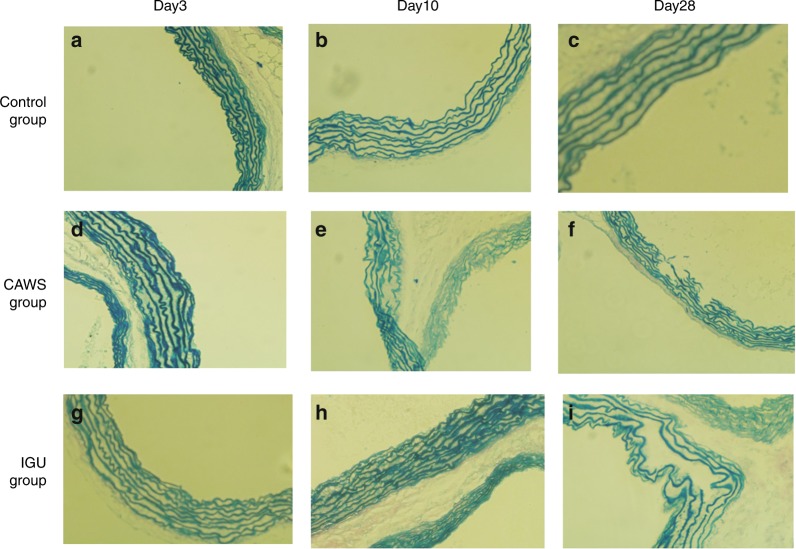
Histopathology of vascular walls after staining the elastic fibers with Verhoeff-van Gieson (EVG) stainin a murine model of vasculitis induced with a *Candida albicans* water-soluble fraction (CAWS). Magnification is at ×100. **a**–**c** Control group; **d–f** CAWS group (layers of vascular walls were in disorder and at day 28, disruption and breakage of elastic fiber was observed); **g–i** iguratimod group (less disorder was observed, and no disruption or breakage was seen). Adapted from Ref., ^91^ copyright © Zhao et al.

Kawasaki disease (KD) is an acute childhood vascular inflammatory disease with an unknown etiology affecting the small-sized to medium-sized coronary arteries.^89^ The disease is characterized by aneurysms, thrombosis, myocardial infarction, embolism, and sudden death.^90^ Iguratimod’s anti-inflammatory potential has been explored by Zhao et al. to treat KD in murine mouse models.^91^ At day 3 after iguratimod’s first administration, the vasculitis was ameliorated, and the effect persisted for 28 days. IL-6 is considered a key serum biomarker for KD; after iguratimod administration, its level was significantly suppressed, and the disease was ameliorated, as confirmed by histopathology of the vessels (Fig. 6).

Refractory lupus nephritis is another condition in which autoimmune reactions take place in the kidney and result in severe nephritis and ultimately kidney failure.^92^ Recently, Yan et al. reported that iguratimod could clinically lower the levels of CD19^+^CD20^-^CD27hi CD38hi cells in plasma.^93^

Moreover, B-cell proliferation was unaffected by iguratimod, whereas B lymphocyte-induced maturation protein-1 (Blimp-1) and X-box binding protein 1 (Xbp-1) signaling pathways were hampered. In addition, the patients treated with iguratimod had a significant reduction in proteinuria at week 8 of treatment. Similarly, Yan et al. also reported that iguratimod could ameliorate autoimmune nephritis and significantly lower proteinuria along with associated markers, such as B-cell-activating factor (BAFF), IL-17A, IL-6, IL-21, anti-dsDNA, and immunoglobulin levels.^94^

#### Cancer-associated conditions

The initial investigations of iguratimod efficacy against cancer metastasis have reported subtle-to-no effect on tumor growth arrest and prevention of its metastases. However, the secondary associated conditions, where interleukins (e.g., IL-6) are involved, are ameliorated. Nevertheless, a recent report from Sakamoto et al. suggested that iguratimod therapy for 6 weeks in a hepatocellular carcinoma xenograft model resulted in lower IL-8 levels, angiogenesis, and number and size of the tumors than those in the control group, suggesting that iguratimod may have some antitumor effects.^95^ Other studies have reported that iguratimod is associated with hepatocellular toxicity in noncancer patients and experimental models.^96^ Therefore, the use of iguratimod to treat hepatocellular carcinoma metastases cannot be overlooked.

**Fig. 7:**
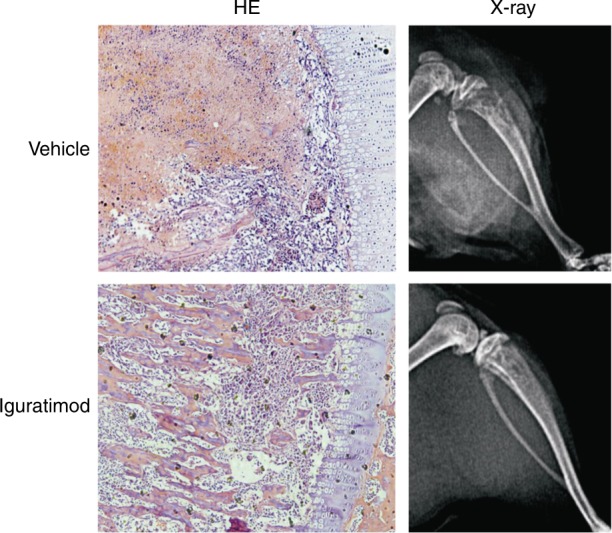
Bone protection during metastasis by iguratimod in a cancer-induced bone destruction rat model. The X-ray radiographs and histopathology lesions after H&E staining (×100 magnification) revealed iguratimod’s ameliorating effects. Adapted from Ref., ^61^ copyright© Huazhong University of Science and Technology and Springer-Verlag Berlin Heidelberg 2016

Sun et al. used iguratimod to alleviate bone degradation during active cancer. They found that iguratimod actively reduced the IL-6 serum level in rat models in an NF-ƙB-dependent manner.^61^ Furthermore, human breast cancer cell lines (MCF-7 and MDA-MB-231) were studied to check their effect on metastasis. Cell proliferation was assessed by Cell Counting Kit-8 (CCK-8) assays and flow cytometry, while migration and invasion properties were evaluated via Transwell and wound-healing assays. Interestingly, a subtle anti-proliferation effect of iguratimod was observed (Fig. 7), whereas anti-migration and anti-invasion effects were not observed.

Cachexia is one of the conditions most commonly associated with cancer^97,98^ and autoimmune diseases, i.e., RA characterized by wasting, weakness, and anorexia.^99^ In adenocarcinoma-induced cachexic mice, the iguratimod effect was evaluated by Tanaka et al.^100^ They found that iguratimod reduced the IL-6 serum level by suppressing its mRNA level, which resulted in inhibition of muscle wasting (gastrocnemius) and loss of adipose tissue (epididymis). However, they did not find any tumor-ablating effect on adenocarcinoma. In addition, they found no promising effect of MTX on cachexia improvement. These different responses provide additional evidence that MTX and iguratimod have a different mode of action.

Recently, Ooka et al. used a chondrosarcoma (OUMS-27) cell line to identify the protein profile effect of iguratimod treatment.^101^ Although chondrosarcoma is a cancer cell line, in this study, they used it for protein profiling that may be beneficial to understanding iguratimod’s mode of action in RA amelioration. The cells were treated with 100 µmol·L^−1^ iguratimod, and the proteins were isolated and then analyzed by mass spectrometry for protein spots of interest. At 24 h, 776 spots were observed, and at day 6, the number was 803. A total of 22 proteins showed a higher intensity, and 15 showed a lower intensity (cutoff values 1.3-fold). Interestingly, heterogeneous ribonuclear protein (hnRNP) A2/B1 and A1 were lower after iguratimod treatment compared with their starting values, and they are highly expressed in the RA synovium in vivo and fibroblast-like synoviocytes in vitro. These proteins are also known as autoantigens of RA that mimic proinflammatory cytokines, e.g., NF-ƙB.

#### Neurodegenerative diseases

Multiple sclerosis is an inflammatory demyelinating disease that affects the central nervous system and is characterized by recurrent episodes of focal demyelinating symptoms. Recently, experimental autoimmune encephalomyelitis (EAE) in rats, which is an experimental model of multiple sclerosis, was ameliorated by iguratimod injections that lowered the levels of TNF-α and IFN-_ϒ_ production and inhibited cellular proliferation in the central nervous system, i.e., the spinal cord.^102^ Similarly, Li et al. also found an ameliorating effect of iguratimod on EAE by significantly reducing demyelination and infiltration of CD169^+^, F4/80^+^ and CD3^+^ cells, Th1 and Th17 helper cells, NF-ƙB p65, and cyclooxygenase expression in the spinal cord. They also observed macrophage/microglia activation suppression in the parenchyma after treatment with iguratimod.^103^

In addition, Bloom et al. used iguratimod to treat experimental autoimmune encephalitis (i.e., a multiple sclerosis model) in synergy with glucocorticoids by protecting the macrophage migration inhibitory factor, whose unique nature is to act as a counter-regulator of the anti-inflammatory effect of glucocorticoids.^104^

Neuropathic pain is also associated with various diseases, including RA and diabetes mellitus. Recently, Morimoto et al. used a chronic constriction injury rat model for neuropathy induced by ligation of the left sciatic nerve to observe the iguratimod effect.^105^ After 15 days of iguratimod administration, the von Frey hair test was used on the hind paw, and histological lesions were observed at the fourth lumbar vertebra region for microglial cells. They found that iguratimod significantly reduced neuropathic pain at 7 days and improved histopathological lesions.

### Iguratimod-associated adverse effects

Thus far, iguratimod has been a welcome alternative where other DMARDs are less responsive, or conventional RA therapy has failed. However, certain adverse effects, including nausea, dizziness, headache, and itching, have been reported as associated conditions with iguratiomd.^15^ In the Chinese population during phase III clinical trials, the incidence of adverse effects in the IGU-treated group was 48.5%, which was almost the same as the widely used RA drug MTX, i.e., 46.1%.^106^ Elevation of liver enzymes has been reported as a significant side effect of iguratimod.^96^ A Japanese clinical study indicated a 5.5% and 9.8% increase in ALT and AST in patients given a combination therapy of IGU and MTX for 24 weeks treatment that further increased to 14.6% and 16.5% at week 52, respectively.^37,77^ The higher incidence of increased aminotransferases is not only unique to IGU but is also common to treatment with MTX and SASP. In the phase III study with the same regimen of IGU, increased transaminase (13.5%) was also the most common adverse effect, while the incidence of increased transaminase in the MTX (MTX 10 mg per week orally for the first 4 weeks and 15 mg per week orally for the subsequent 20 weeks) group was observed to be 23.9%.^106^ Hence, liver function monitoring during treatment with IGU is recommended.

Iguratimod has also been reported to have a higher risk of hemorrhage after combination therapy with warfarin. VK-dependent blood coagulation factors have been demonstrated to be involved in the iguratimod and warfarin interaction.^107^ The author’s personal clinical experience has demonstrated high blood pressure and skin rashes to be adverse effects of iguratimod in a few patients. However, during clinical trials, the participants exhibited neither gastric ulcers during gastric endoscopy nor cardiovascular ailments during circulatory system monitoring.^106^

Recently, a Japanese study reported the results of a postmarketing surveillance of 2 679 patients after 24 weeks of treatment with IGU. The authors reported IGU to be safe and effective without any life-threatening adverse effects.^108^

### Outlook and conclusion

Iguratimod is a welcome addition to the DMARD family for the treatment of autoimmune diseases and amelioration of associated inflammation. Thus far, the overall efficacy of iguratimod in clinical RA and bone protection is satisfactory, whereas its associated adverse effects need to be closely monitored.

Moreover, it can be used as a coating on the surface of biomaterials that are intended to be used in prosthetic surgery, e.g., orthopedic implants, orthodontics, maxillofacial surgeries, and cardiovascular stents. Iguratimod may not only reduce the biomaterial-associated inflammation but also lower their rejection rate, which is one of the most common problems with prosthetic implants.

In summary, we can conclude that iguratimod is a relatively new DMARD, primarily used to ameliorate RA via suppression of cytokines and immunoglobulins, i.e., IL-6, TNF-α, IL-17, and the NF-ƙB pathway. To better reduce its adverse effects, we may use 25 mg per day for an initial 4 weeks and 50 mg per day after that. Moreover, its combination with MTX or monotherapy was proven to be highly synergistic, especially in combination with MTX and other DMARDs for nonresponding refractory patients.
